# Performance et coût de la prise en charge de la malnutrition aiguë sévère avec complications à Kaya, Burkina Faso

**DOI:** 10.11604/pamj.2019.34.145.17946

**Published:** 2019-11-14

**Authors:** Bassibila Zoungrana, Prosper Saga Sawadogo, Namwin Siourimè Somda, François Tapsoba, Abel Tankoano, Aly Savadogo

**Affiliations:** 1 Centre de Recherche en Sciences Biologiques, Alimentaires et Nutritionnelles, Laboratoire de Biochimie et Immunologie Appliquées (CRSBAN/LaBIA), Unité de Formation et de Recherche en Sciences de la Vie et de la Terre (UFR/SVT), Université Ouaga I Pr Joseph Ki-Zerbo, 03 BP 7021 Ouagadougou 03, Ouagadougou, Burkina Faso; 2 Food and Agriculture Organization of United Nations (FAO), Ouagadougou, Burkina Faso

**Keywords:** Emaciation, traitement, coût, Burkina Faso, Emaciation, treatment, cost, Burkina Faso

## Abstract

**Introduction:**

Les conséquences de la malnutrition aiguë sévère s’expriment en termes de santé et de survie de la personne affectée, mais aussi en termes de développement intellectuel de l’individu, sa productivité et globalement l’économie nationale. Sa prise en charge nécessite d’énormes moyens financiers. L’objectif de la présente étude est d’évaluer la performance de la prise en charge *versus* coût du traitement des enfants malnutris aigus sévères.

**Méthodes:**

C’est une étude rétrospective réalisée de janvier à décembre 2014 auprès de 199 enfants de 0 à 59 mois admis au centre de récupération et d’éducation nutritionnelle de Kaya, Burkina Faso. Le coût de la prise en charge, la durée de séjour, le gain de poids journalier et la vitesse de récupération ont été analysées selon les méthodes de calcul standards. Le test de Mann-Whitney et de Kruskall-Wallis ont été utilisés pour comparer les médianes au seuil de 0,05.

**Résultats:**

Comme présagé, les enfants de 6 à 23 mois étaient les plus affectée (51,8 %) et les infections respiratoires aiguës étaient les pathologies les plus associées (57,9 %). La durée médiane de séjour était de 9,0 (7,0 - 13,0) jours, la vitesse de récupération médiane de 100,0 (65,8 - 143,3) g/j et le gain pondéral médian journalier de 18,1 (11,6 - 27,7) g/kg/j. Le coût moyen de traitement d’un enfant malnutri est estimé à 15715,3 FCFA (25,2 USD).

**Conclusion:**

Le coût de traitement est difficilement supportable par les parents des enfants malnutris d’où la nécessité de l’intervention du gouvernement et ses partenaires au développement.

## Abstract (en)

**Introduction:**

The consequences of severe acute malnutrition are measured in terms of health and survival, but also of cognitive development, its productivity and the overall national economy. Its management requires enormous financial resources. The purpose of this study was to assess the effectiveness of treating severe acute malnutrition versus cost of treatment of severe acute malnutrition in children.

**Methods:**

We conducted a retrospective study of 199 children aged 0-59 months admitted to the Centre for Nutritional Recovery and Education in Kaya, Burkina Faso, from January to December 2014. The cost of treatment, the length of stay in the Centre for Nutritional Recovery and Education, daily weight gain and the speed of recovery were analyzed based on the standards calculation methods. Mann-Whitney test and Kruskall-Wallis test were used to compare the medians (0.05 threshold).

**Results:**

As expected, children aged 6-23 months were the most affected (51.8%) and acute respiratory infections were the most associated diseases (57.9%). The median length of stay in the Centre for Nutritional Recovery and Education was 9.0 (7.0-13.0) days, the mean speed of recovery was 100.0 (65.8 - 143.3) g/day and the average daily weight gain was 18.1 (11.6 - 27.7) g/kg/day. The average cost of treatment in a malnourished child is estimated to be 15 715,3 FCFA (25.2 USD).

**Conclusion:**

The cost of treatment is hardly affordable by the parents of malnourished children; hence the necessity for government and development partners interventions.

## Introduction

La malnutrition constitue un problème de santé publique dans le monde, pouvant remettre en cause les objectifs de développement durables [1], notamment dans les pays en développement. Elle revêt de nombreuses formes: des enfants et des adultes qui n’ont que la peau sur les os, des enfants qui ne grandissent pas convenablement, des individus en mauvaise santé à cause de carences de nutriments dans leur alimentation, des personnes obèses ou atteintes de maladies non transmissibles liées à l’alimentation; touche tous les pays et une personne sur trois dans le monde [1, 2]. Au Burkina Faso, le rapport de l’enquête nutritionnelle nationale a montré que la prévalence de la malnutrition aiguë chez les enfants de 0 à 59 mois était de 7,6% avec 1,4% de forme sévère [3]. Une seule région présentait une prévalence de malnutrition aiguë supérieure au seuil critique de 10% établit par l’OMS [3]. Les enfants malnutris aigus sévères avec complications sont admis en interne pour être stabilisés conformément au protocole national de prise en charge. Cette prise en charge s’effectue en différentes phases nécessitant ainsi d’énormes coûts financiers. Cependant, au Burkina Faso, il existe très peu de données sur le coût du traitement de la malnutrition aiguë d’où l’intérêt de la présente étude. L’étude vise à décrire les principaux déterminants de performance et à déterminer le coût de la prise en charge des enfants malnutris aigus sévères avec complications.

## Méthodes

Il s’est agi d’une étude rétrospective basée sur la revue documentaire comprenant les activités quotidiennes de janvier à décembre 2014 enregistrées dans la fiche de suivi thérapeutique des enfants. L’étude a eu pour cible les enfants de 0 à 59 mois admis au Centre de Récupération et d’Education Nutritionnelle (CREN) du Centre Hospitalier Régional (CHR) de Kaya, Burkina Faso. Elle a concerné 199 enfants ayant souffert de la Malnutrition Aiguë Sévère avec Complications (MASaC) traitées avec succès. Ces enfants ont été choisis de façon aléatoire simple parmi 512 enfants malnutris guéris au cours de l’année 2014 à l’aide du logiciel Emergency Nutrition Assessment for SMART 2011. Etait éligible tout enfant ayant souffert de la MASaC admis au CREN et traités avec succès pour ses complications. Les variables étudiées étaient des paramètres sociodémographiques (l’âge et le sexe de l’enfant); des paramètres anthropométriques (le poids, la taille, le périmètre brachial) et les complications associées à l’admission de l’enfant malnutris.

Les principaux indicateurs de performance ont été calculés selon les formules suivantes définies dans le protocole de prise en charge de la malnutrition au Burkina Faso présentes dans le Tableau 1. Les critères de classification par rapport au gain pondéral médian journalier est: gain pondéral médian journalier faible (< 5 g/kg/j), gain pondéral médian journalier modéré (5-10 g/kg/j) et gain pondéral médian journalier satisfaisant (> 10 g/kg/j) [4].

**Tableau 1:** Principaux indicateurs de performance

Paramètres	Abréviation du Paramètre	Unités	Formules
Durée de séjour	DS	j	= (Date de sortie (j) – Date d’admission (j))
Gain de poids journalier	GPJ	g/kg*j	= (Poids de sortie (g) – Poids minimum (g)) / (Poids minimum (kg) * Nombre de jour entre la date du poids minimum et la date de sortie (j))
Vitesse de récupération	VR	g/j	= (Poids de sortie (g) – Poids minimum (g)) / (Nombre de jour entre la date du poids minimum et la date de sortie (j))
Coût moyen de la PEC de la MASaC	CM-PEC-MASaC	F CFA	= Somme des dépenses effectuées au cour du traitement des patients guéris / Nombre total de patients guéris

Les données ont été saisies à l’aide du logiciel Epi Info 5.3.1 puis les analyses statistiques ont été réalisées à l'aide du logiciel SPSS version 21.0 pour la détermination de la moyenne, son erreur standard ou de la médiane, son intervalle interquartiles et du pourcentage (%). Le test U non paramétrique de Mann-Whitney a été utilisé pour tester l'égalité de deux médianes. La comparaison de plus de deux médianes a été effectuée par le test non paramétrique de Kruskall-Wallis ANOVA à un facteur au seuil de significativité de 0,05.

## Résultats

L’étude a montré que la proportion des garçons (50,3%) était comparable à celle des filles (49,7%). La tranche d’âge de 6 à 23 mois était la plus affectée par la malnutrition et représentait plus de la moitié de l'échantillon (51,8%). L’âge médian des enfants malnutris était de 12,0 (6,0; 24,0) mois. Les pathologies les plus associées à la MASaC étaient: les infections respiratoires aiguës (57,9%), l’anémie (25,3%) et la diarrhée (23,2%). De plus, la malnutrition avec œdèmes représentait 21,1% des cas.

La durée médiane de séjour des enfants sortis guéris était de 9,0 (7,0; 13,0) jours avec des extrêmes de 2 à 40 jours. Elle variait significativement en fonction de la présence ou de l’absence d’œdèmes bilatéraux et de la présence d’une ou de plusieurs comorbidités associées. La vitesse de récupération médiane de cette étude a été de 100,0 (65,8; 143,3) g/j. Elle avait une différence significative en fonction de l’âge et du poids minimum. Le gain pondéral médian journalier était de 18,1 (11,6; 27,7) g/kg/j. Il a varié significativement en fonction de l'âge, de la présence d'œdèmes, de la présence de plusieurs comorbidités associées et du poids minimum. Le coût moyen de la prise en charge des enfants MASaC au CREN du CHR de Kaya a été estimé à 15715,3 FCFA (25,2 USD) avec un écart type de 8667,3 FCFA (13,9USD).

## Discussion

### Biais de l’étude

La cible de l’étude ayant été les enfants MASaC guéris, nous avons été confronté aux manques de données lorsque l’enfant avait un état très grave à l’admission et ayant commencé son traitement aux urgences. Ainsi, certaines données surtout les traitements médicaux de ses enfants n’ont pas toujours été reportés sur la fiche de suivi de prise en charge thérapeutique.

### Aspects épidémiologiques

La malnutrition dans cette partie du Burkina concerne à la fois les jeunes garçons et les jeunes filles avec des proportions respectives de 50,3% et de 49,7%. Cette étude montre que le sexe de l’enfant de moins de 5 ans n’est pas un déterminant de son état de santé nutritionnelle contrairement à plusieurs études antérieurs [5-7]. En effet, Ouédraogo [8] et Dembélé [9] ont rapportés une prédominance de la malnutrition aiguë sévère chez les jeunes filles comparativement aux jeunes garçons. La tranche d’âge de 6 à 23 mois était la plus affectée par la malnutrition aiguë sévère (*p* = 0,000) et représentait plus de la moitié de l'échantillon soit 51,8%. Cette tranche d’âge a été reconnue par plusieurs auteurs comme étant un âge de prédilection pour la survenue de la malnutrition [10-12]. En effet, c'est pendant cette période de la vie qu'intervient la diversification alimentaire. La malnutrition à cet âge pourrait s’expliquer par une diversification alimentaire inadéquate, faite précocement ou tardivement ou avec des aliments de complément non adaptés, ou une ablactation précoce suite à la survenue d’une nouvelle grossesse. Elle pourrait également être la résultante de la consommation insuffisante des aliments, de mauvaise pratique des règles d’hygiènes alimentaires et/ou une mauvaise fréquentation des services de santé.

### Aspects cliniques

Les pathologies les plus associées à la MASaC étaient: les infections respiratoires aiguës (57,9%), l’anémie (25,3%) et la diarrhée (23,2%). La prévalence de la malnutrition associée aux infections respiratoires aigües trouverait son explication dans le fait que la malnutrition est responsable d’une défaillance du système immunitaire du patient, ce qui le rend vulnérable aux infections. Cette prévalence est supérieure à celles obtenues par Barry [13] et Ouédraogo [14] avec des prévalences respectives de 24,40% et 27,45%. L’association de la malnutrition aiguë sévère (MAS) et de l’anémie; est présente chez 25,3% d’enfants malnutris. Cette prévalence élevée de l’anémie pourrait être non seulement d’origine nutritionnelle, infectieuse, mais aussi d’origine palustre du fait de l’endémie du paludisme que connaît la région du Centre Nord du Burkina Faso. Ainsi, au cours de l’étude, 18,4% de cas de paludisme associé à la malnutrition aiguë sévère ont été enregistrés. La prévalence de la MAS associée à l’anémie était inférieure à 32,10% rapportée par Barry [13]. Quant à la diarrhée, elle est fréquemment rencontrée dans la malnutrition aiguë sévère, du fait des parasitoses, des infections bactériennes ou virales et de la malabsorption; elle était présente chez 23,2% des enfants malnutris aigus sévères. Cette prévalence est similaire à celle obtenue par Barry [13] avec une prévalence de 23,10% mais, elle est inférieure à ceux de Ouédraogo [14]; Zoubga [6] et Félicitée *et al.* [15] avec des prévalences respectives de 62,90%; 41,20% et 34,00%.

### Durée médiane de séjour, vitesse de récupération médiane journalier et gain pondéral médian journalier

Dans cette étude, la durée médiane de séjour était de 9,0 (7,0; 13,0) jours (Tableau 2). Elle variait significativement en fonction de la présence ou d’absence d’œdèmes bilatéraux (*p* = 0,012) et de la présence d’une ou de plusieurs comorbidités associées (*p* = 0,034). En effet, pendant la phase de réhabilitation nutritionnelle, les malnutris avec œdèmes connaissent tout d'abord une perte pondérale résultante à la fonte des œdèmes. L'ascension pondérale ne survient que par la suite. Cependant, les malnutris sans œdèmes gagnent directement du poids dès la phase de réhabilitation nutritionnelle. Ceci expliquerait le long séjour dans la malnutrition avec œdème que dans celle sans œdème. Par ailleurs, les comorbidités contribuent grandement à l'allongement de la durée de séjour des malnutris. Ainsi, les malnutris avec plusieurs pathologies associées connaissaient un séjour plus long que ceux avec une pathologie associée. L’allongement de la durée médiane de séjour dû à la présence des œdèmes nutritionnels et à la présence de plusieurs comorbidités a été également souligné par Richard *et al.* [7].

**Tableau 2:** Durées médianes de séjour des enfants admis au CREN en fonction de leurs caractéristiques sociodémographiques, cliniques et anthropométriques

Variables	Médiane (EIQ) (jours)	N	P
	9 (7,0; 13,0)		
**Sexe**		**199**	0,317
Masculin	9,0 (7,0; 12,0)	100	
Féminin	9,0 (7,0; 14,0)	99	
**Age (mois)**		**199**	0,112
< 6	8,0 (7,0; 12,2)	44	
6 à 11	9,0 (7,0; 12,0)	42	
12 à 23	9,0 (6,0; 12,8)	59	
24 à 59	10,0 (8,0; 15,0)	52	
**Œdèmes bilatéraux**		**199**	**0,012**
Oui	11,0 (8,0; 15,0)	40	
Non	9,0 (7,0; 12,0)	159	
**Comorbidités associées**		**190**	**0,034**
Une comorbidité	8,0 (6,5; 12,0)	61	
Plusieurs comorbidités	10,0 (8,0; 13,5)	129	

La vitesse de récupération médiane de 100,0 (65,8; 143,3) g/j est fonction du poids minimum du (*p* = 0,000) et de l’âge (*p* = 0,000), les plus jeunes enfants présentant la vitesse de récupération la plus faible (Tableau 3). L'autonomie alimentaire des enfants plus âgés leur permettant de se nourrir plus suffisamment pourrait expliquer cette situation. Le gain pondéral médian journalier était de 18,1 (11,6; 27,7) g/kg/j (Tableau 4). Il a varié significativement en fonction de l'âge (*p* = 0,000), de la présence d'œdèmes (*p* = 0,004), de la présence de plusieurs comorbidités associées (*p* = 0,024) et du poids minimum (*p* = 0,000). Il était rapide chez les enfants malnutris sans œdèmes et chez ceux ayant une seule pathologie par rapport aux enfants malnutris œdémateux et associés à plusieurs pathologies. En outre, ce gain était plus élevé chez les enfants de moins de 06 mois et ceux de poids minimum inférieur à 3,5 kg comparativement aux enfants de 06 à 23 mois et plus et aux enfants de plus de 3,5 kg. En effet, chez les enfants de 0 à 24 mois, la vitesse de croissance est très rapide, alors qu'elle se ralentit entre deux ans et le début de la puberté [16]. Richard *et al.* [7] ont également rapportés que l’âge était un facteur déterminant du gain pondéral médian journalier de l’enfant malnutris, mais ont noté une absence de variation significative du gain pondéral médian journalier en fonction des œdèmes nutritionnels et des comorbidités associés. Le gain pondéral médian journalier observé au cours de cette étude (18,1 g/kg/j) est conforme, gain satisfaisant de 10 à 20 g/kg/j nécessaire pour une bonne réhabilitation nutritionnelle [17]. Ces résultats sont confirmés par les travaux d’Ashworth *et al.* [4] qui montrent qu'une réhabilitation nutritionnelle des enfants malnutris est satisfaisante si le gain pondéral quotidien est supérieur à 10 g/kg/j. Il est supérieur à ceux rapportés en Tanzanie (15,0 g/kg/j) [18], au Sénégal (7,6 g/kg/j) [19] et en République Démocratique du Congo (7 g/kg/j) [7].

**Tableau 3:** Vitesses de récupération médiane journalière des enfants malnutris en fonction de leurs caractéristiques sociodémographiques, cliniques et anthropométriques

Variables	Médiane (EIQ) (gramme/jour)	N	P
	100,0 (65,8; 143,3)		
**Sexe**		**197**	0,753
Masculin	100,0 (69,1; 139,4)	100	
Féminin	100,0 (63,6; 150,0)	97	
**Age (mois)**		**197**	**0,000**
< 6	77,8 (48,1; 100,0)	44	
6 à 11	86,9 (62,5; 121,3)	42	
12 à 23	117,7 (84,6; 150,0)	59	
24 à 59	117,4 (84,4; 187,7)	52	
**Œdèmes bilatéraux**		**197**	0,172
Oui	116,7 (75,0; 157,1)	39	
Non	100,0 (64,3; 140,7)	158	
**Comorbidités associées**		**188**	0,444
Une comorbidité	100,0 (62,9; 126,7)	60	
Plusieurs comorbidités	100,0 (66,7; 150,0)	128	
**Poids minimum (kilogramme)**		**197**	**0,000**
< 3,50	70,0 (46,7; 92,9)	35	
3,50 à 6,99	100,0 (64,3; 127,5)	97	
7,00 à 15,00	120,0 (87,5; 187,6)	65	

**Tableau 4:** Gain pondéral médian journalier au cours du séjour des enfants malnutris en fonction de leurs caractéristiques sociodémographiques, cliniques et anthropométriques

Variables	Médiane (EIQ) (gramme/kilogramme/jour)	N	P
	18,1 (11,6; 27,7)		
**Sexe**		**197**	0,859
Masculin	18,1 (12,1; 27,6)	100	
Féminin	18,1 (11,2; 27,8)	97	
**Age (mois)**		**197**	**0,000**
< 6	29,6 (21,9; 40,5)	44	
6 à 11	16,7 (11,0; 22,1)	42	
12 à 23	17,2 (12,1; 23,1)	59	
24 à 59	14,0 (9,9; 22,7)	52	
**Œdèmes bilatéraux**		**197**	**0,004**
Oui	13,5 (9,3; 19,2)	39	
Non	19,9 (12,8; 27,9)	158	
**Comorbidités associées**		**188**	**0,024**
Une comorbidité	21,2 (13,5; 33,4)	60	
Plusieurs comorbidités	16,7 (11,1; 25,0)	128	
**Poids minimum (kilogramme)**		**197**	**0,000**
< 3,50	30,8 (23,3; 19,2)	35	
3,50 à 6,99	17,8 (11,3; 23,1)	97	
7,00 à 15,00	14,5 (10,9; 22,0)	65	

### Coût moyen de la Prise En Charge (PEC) en hospitalisation

Le coût moyen de la PEC des enfants MASaC au CREN du CHR de Kaya a été estimé à 15715,3 FCFA (25,2 USD) avec un écart type de 8667,3 FCFA (13,9 USD) (Tableau 5). Cet écart type très grand pourrait être dû au fait que la prise en charge de certains enfants a été faite à des prix très réduites alors que celle d’autres enfants ont été faite à des prix très élevés. En effet, le minimum et le maximum du coût de la prise en charge étaient respectivement de 2575,4 et 58499,4 FCFA pour une durée de séjour de 5 et 40 jours. Il y’a donc une étroite relation entre le coût de la prise en charge et la durée de séjour. Cette corrélation a été également soulignée par Ouédraogo [14]. Le coût moyen de la prise en charge de la MASaC serait difficilement supporté par les parents dont la majorité vivait en milieu rural. En effet, 40,1% de la population burkinabè vit en dessous du seuil de la pauvreté et la grande proportion de ses pauvres vit en milieu rural [20]. Ainsi, en l’absence d’exemption de payement des frais de la prise en charge, ce coût élevé provoquerait la désertion des services de santé, pouvant accroitre le phénomène de la malnutrition dans cette localité du Burkina Faso.

**Tableau 5:** Coût moyen de la PEC* de la MASaC** par tranches d’âge et par produits au CHR*** de Kaya

Différents Prix (Franc CFA)	< 6 mois			6 à 59 mois		
	Effectifs des enfants	Prix total	Prix Moyen	Effectifs des enfants	Prix total	Prix moyen
ReSoMal ^4^*	8	46,7	5,8 ± 02,0	36	332,8	09,2 ± 02,7
SNG^5^*	3	300,0	100,0 ± 00,0	66	19 400,00	293,9 ± 245,5
©Plumpy’Nut	2	11 692,6	5 846,3 ± 4 452,0	147	515 073,6	3 503,9 ± 2 015,1
Lait 1^re^ âge	25	80 000	3 200,0 ± 00,0	0	-	-
Lait F100Dilué	21	58 469,5	2 784,3 ± 1 224,5	0	-	-
Lait F75	2	3 879,4	1 939,7 ± 1 269,2	155	995 120,1	6 420,1 ± 3 690,3
Lait F100	0	-	-	88	407 770,5	4 633,8 ± 4 026,0
Traitement systématique et examen	41	165 604,7	4 039,1 ± 4 021,4	146	793 452,1	5 434,6 ± 4 646,8
Total	41	310 782,5	**7 580,1 ± 4 368,1**	146	2 569 354,2	**17 598,3 ± 8 518,4**
Prix en Franc CFA (USD)	**0 à 59 mois**					
	**Effectifs des enfants**		**Prix total**		**Prix moyen**	
Coût PEC* MASaC**	**187**		**2 938 755,7 (4 711,3)**		**15 715,3 ± 8 667,3 (25,2 ± 13,9)**	

* PEC : Prise En Charge ;

** MASaC : Malnutrition Aiguë Sévère avec Complications ;

*** CHR : Centre Hospitalier Régional ;

**** ReSoMal : *Rehydratation Solution for Malnutrition ;* ***** SNG : Sonde Nasogastrique

## Conclusion

L’étude a porté sur 199 enfants malnutris de 0 à 59 mois avec un âge médian de 12,0 mois dont la tranche d’âge la plus touchée était ceux de 6 à 23 mois soit 51,8%. La pathologie la plus fréquemment associée était les infections respiratoires aigües (57,9%). La prise en charge a été correctement faite. Cependant, le traitement médical de 12 enfants malnutris n’ont pas été répertoriés. La réhabilitation nutritionnelle était satisfaisante avec un gain pondéral médian journalier de 18,1 g/kg/j et une durée médiane de séjour de 9,0 jours. La présence ou l’absence des œdèmes, l’existence d’une ou plusieurs comorbidités s’avèrent être des facteurs déterminants de la durée de séjour et du gain pondéral. Le coût moyen de la prise en charge de la MASaC a été estimé à 15715,3 ± 8667,3 FCFA (25,2 ± 13,9 USD). Ce coût serait difficilement supportable par les parents dans un pays comme le Burkina Faso dont 40,1% de la population vit sous le seuil de la pauvreté. Cependant, plusieurs régions du Burkina Faso ne bénéficiaient pas de la gratuité des soins en 2014. Toutefois, la gratuité des soins pour les enfants de moins de 5 ans instaurée en 2016 par le gouvernement se présente comme un soulagement pour toute la population.

### Etat des connaissances actuelles sur le sujet

- La malnutrition constitue un problème de santé publique;
- Les conséquences de la malnutrition s’expriment en termes de santé et de développement;
- La prise en charge de la malnutrition nécessite des moyens financiers.

### Contribution de notre étude à la connaissance

- Le coût de traitement de la malnutrition est difficilement supportable par les parents;
- Les enfants de 6 à 23 mois étaient les plus concernés;
- Le coût moyen de traitement d’un enfant malnutri est connu.

## Conflits d’intérêts

Les auteurs ne déclarent aucun conflit d’intérêts.

## Remerciements

Les auteurs remercient le personnel administratif et tout le personnel de la pédiatrie du CHR de Kaya pour leurs soutiens multiformes lors de la collecte des données.
